# Gender aspects in allergies of pets – A secondary publication and update

**DOI:** 10.1186/s40413-017-0172-1

**Published:** 2017-12-27

**Authors:** Ina Herrmann, Lukas Einhorn, Lucia Panakova

**Affiliations:** 1 Comparative Medicine, The interuniversity Messerli Research Institute, University of Veterinary Medicine Vienna, Medical University Vienna and University Vienna, Vienna, Austria; 2 Clinics of Small Animals and Horses, University of Veterinary Medicine Vienna, 1210 Vienna, Austria; 3 Institute of Pathophysiology and Allergy Research, Center of Pathophysiology, Infectiology and Immunology, Medical University of Vienna, Vienna, Austria

**Keywords:** Atopic dermatitis, Canine, Cat, Dog, Gender, Horse, Pet

## Abstract

Allergies need not only affect humans; this multifactorial and complex disease can also affect animals. Comparative allergology investigates the many similarities between the pathogenesis, clinics, diagnosis, and therapy of the disorders in humans and pet animals. In contrast to human allergy research, the veterinary field lacks access to a central database, which means there are no cohort studies published. This limits not only the research on breed and regional differences in allergies, but also further studies on the impact of gender in allergies of domestic animals. Moreover, domestic cats, dogs and male horses are castrated in most cases, which neutralises any effects of sexual hormones. In this review article a few interesting findings regarding gender aspects in companion animals were extracted from current literature. In summary, there is a lack of data on gender effects on allergies in cats, dogs or horses.

## Background

Comparative medicine is an interdisciplinary field in which researchers transfer knowledge from the basic research into clinical implications to improve the health of humans and animals. The dog is known as a spontaneous model for atopic dermatitis and many of our companion animals exhibit spontaneous signs of allergic diseases [1]. Moreover, our pet animals are often neutered or spayed to prevent reproduction and/or diseases or to influence their behavior. In this way, some effect of sex hormones on the allergic diseases in this population can be explored. Even if the databases in veterinary medicine are not extensive yet, there is data and some evidence of the influence of sex hormones on the allergic diseases. The purpose of this review was to collect the current evidence of the gender aspects in dogs, cats and horses and their impact on the developement of the allergic diseases.

## Gender distribution of allergies affecting dogs: advances in evidence of greater risk in females?

Canine atopic dermatitis (CAD) is the most investigated and most common allergic disease in companion animals. Due to its similarities to human atopic dermatitis, comparative allergology investigates and compares CAD and atopic disease in humans [1]. In contrast to human medicine, veterinary medicine is far behind with regard to both centralized databases and broad access to medical records of populations of affected animals, or any standardization of allergen testing.

**Fig. 1:**
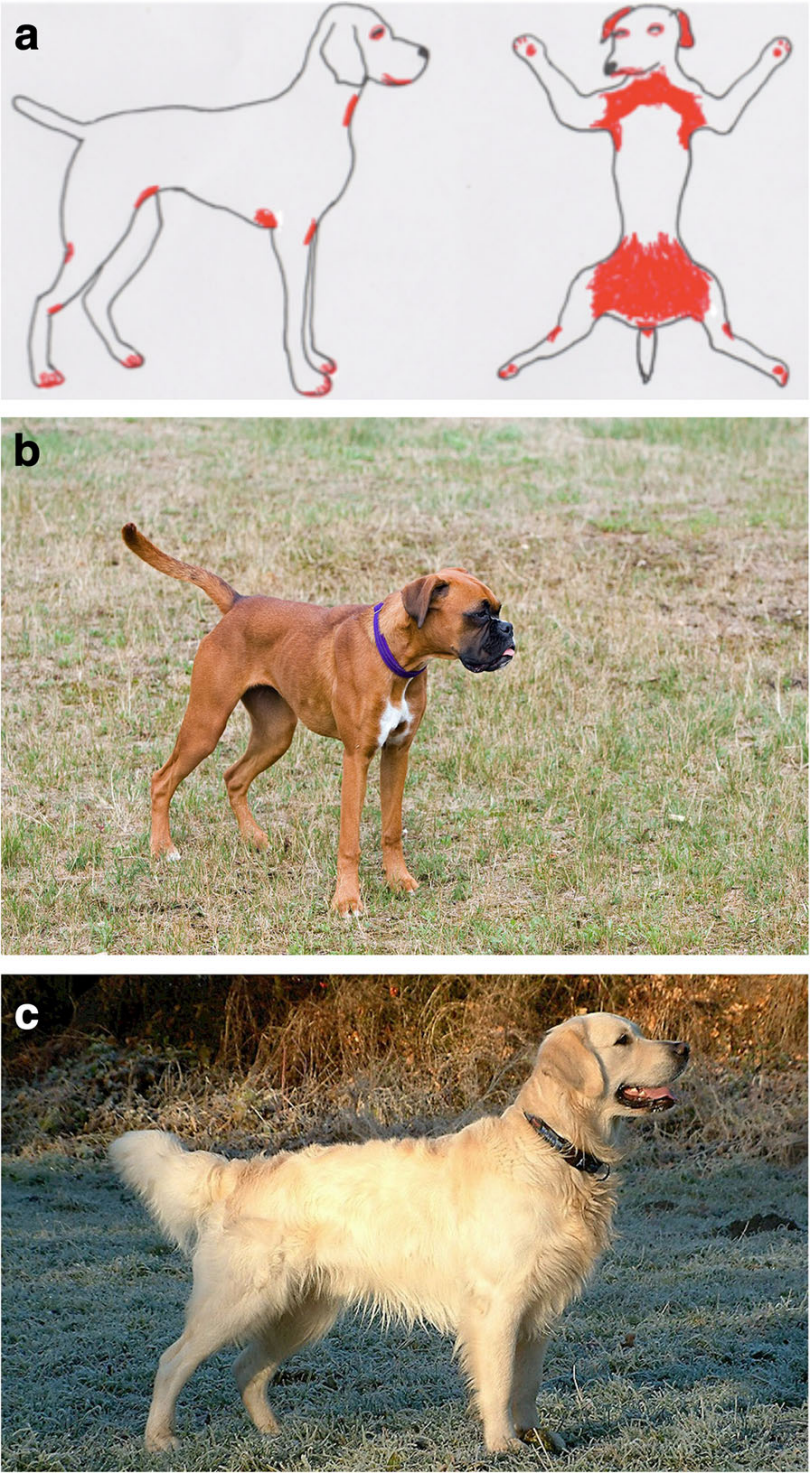
Dogs typically show allergy-related symptoms on their skin. **a**) typical distribution of canine atopic dermatitis (CAD) symptoms (especially erythema, papule and pruritus) in the areas marked with red. A gender predisposition for both **a b**) boxer and **c**) golden retriever has been described; however, further studies are necessary for its confirmation. (Pictures by Pixabay, under the creative commons license on www.pixabay.com and http://commons.wikimedia.org/)

Dogs affected by CAD most frequently exhibit skin lesions (erythema, micropapules) and pruritus on typical body areas - similar to human atopic dermatitis (Fig. 1a) due to a TH2 immune response [2]. In 2001, the ACVD Task Force on Canine Atopic Dermatitis collected and published data regarding this disease and reported contradictory results on the gender distribution [3]. A clinical study from 1981 described a higher prevalence in female dogs (2.5 times increased risk) [4], although a 1983 study showed no gender differences [5]. The conclusion of the ACVD Task Force at the time was that this issue is still unresolved and caution should be taken due to variances in the inclusion criteria caused by a non-standardized approach to diagnosing atopic dermatitis in dogs. The next detailed review paper on CAD written by members of the International Committee on Allergic Diseases in Animals was published in 2015. Their conclusion was based on seven clinical studies published between 2006 and 2012 and suggested that CAD, in general, does not exhibit sex predilection [6]. The biggest article among these studies which included 843 atopic dogs could show no difference between male and female dogs [7]. As with studies from other continents, none could provide any indications of gender disparity [8–10], although two breed-specific exceptions were described in a study by Wilhelm et al. [10], showing a higher number of female boxers and male golden retrievers in the studied population [Fig. 1b, c]. On the contrary, a study from Sweden revealed no sex predisposition in boxer, West Highland white terrier or bullterrier [11]. In most studies a mixed population of affected breeds is investigated. There are only few large studies investigating one dog breed with the aim to prove breed-specific phenotypes of CAD. In two genome-wide linkage studies of West Highland white terrier and German shepherd dogs with CAD, data about the male-female ratio is not included [12, 13], which is symbolic of the low interest in this kind of data. A study focusing on Labrador retrievers and golden retrievers once again showed no influence of the sex on the prevalence of CAD [14].

An extreme bias in most of these previously described studies is caused by the fact that companion animals are often neutered, although regional differences occur. In 2016, a retrospective study evaluated the effect of gonadectomy on immune-mediated diseases in dogs and for the first time revealed some interesting insights into this topic [15]. Under the investigated diseases, CAD was the most common disease in 90.090 dogs, presented between 1995 and 2010 to a veterinary teaching hospital in California. In the population of 1638 dogs with CAD, 83 were intact females, 745 neutered females, 169 intact males and 641 neutered males, resulting in a greater relative risk of disease in both neutered sexes. In addition, neutered females were at a 1.5–2 fold higher danger of developing atopic dermatitis [15]. A possible explanation is the role of gonadal steroids on the immune response, especially on the TH2 type hypersensitivity. Estradiol seemed to accelerate the progression of humoral immune response via enhancing the TH2 pathway, while androgen has a protective effect [16]. These findings could indeed explain the results in the California study, but more data is required to suggest any clear evidence for a gender impact on CAD. Nevertheless, dogs present an interesting model of evaluation of the gender aspect in AD due to their classification as non-neutered female and male, and neutered female and male animal groups.

Interestingly enough, even in human literature where significantly more data from birth-control studies, cohort studies and central databases are available, the evidence for a clear gender difference in AD is conflicting [17]. On the other hand, even if human atopic dermatitis (AD) and CAD are compatible in regard to the pathogenesis and clinical presentation, their treatment differs considerably. Causative allergens can be found in most of the dogs with CAD (intradermal test and/or serum IgE test), and in most of the treated dogs, allergen-specific immunotherapy (ASIT) reduces the symptoms [18]. Therefore, a direct comparison should be made carefully.

Other allergies in dogs are flea allergy dermatitis (FAD) and food induced atopic dermatitis (FIAD). At the moment, FIAD is considered to be the same disease as CAD and its main clinical symptoms are comparable to CAD. Only the trigger allergens differ, hence the name food induced atopic dermatitis (FIAD). Although in theory the influence of sex hormones should have an impact on the pathogenesis, there is currently no evidence of gender differences present in FIAD and FAD studies [19, 20].

## Female gender predispositions for hypersensitive dermatitis in cats

As is the case with dogs, cats are not registered in a central database system either, with the exception of Switzerland. Central registration of cats, along with systemic data collection by veterinarians, would facilitate cohort population studies in feline allergology and dermatology.

Not unlike dogs, cats frequently show allergic skin symptoms that necessitate a visit at veterinary clinics. Due to the decrease in investigation into the pathogenesis of allergies in cats, the nomenclature of allergic skin diseases in felines has yet to be standardized. Additionally, limited diagnostic methods for allergic feline diseases contribute to a confusing nomenclature, which makes differentiating the three main allergic skin diseases a complex problem. Consequently, queries in medical databases (keywords: feline/cat Atopy, feline/cat Atopic Dermatitis, cat/feline Allergic Dermatitis and Dog/Canine Atopic Dermatitis, Dog/Canine Atopy and Dog/Canine Allergic Dermatitis) result in 4 times fewer references to studies on feline allergies compared to studies on canines.

In contrast to dogs, cats with allergic skin diseases show comparatively unspecific symptoms – the so-called feline cutaneous reactive pattern seen in veterinary dermatology which includes:

1. Itching and excoriations on the head and neck area (head and neck pruritus)
2. Symmetrical self-induced ventral alopecia
3. Eosinophilic granuloma complex (eosinophilic granuloma, eosinophilic plaques and indolent/eosinophilic ulcer)
4. Miliary dermatitis and others.

Feline cutaneous reactive patterns are neither pathognomonic of environmental allergies (atopic dermatitis, or "non-flea, non-food hypersensitivity disorder", [NFNFHD]), nor specific to allergies in cats in general (flea allergy, food allergy and atopic dermatitis — NFNFHD). The field of research that is focused on feline allergies lacks bigger cohort studies, as in the past only small studies (with a few patients) and studies in restricted geographical areas have been published.

In terms of the influence of sexual hormones on the development, duration and severity of feline atopic dermatitis, the following aspects have to be considered. Regardless of sex, the majority of cats in western countries, where most of the published studies about atopic dermatitis or the eosinophilic granuloma complex in cats are being carried out, are castrated early (at 4–12 months of age). Only a few individuals, usually pedigree cats used for breeding, remain intact. As a consequence, less information about family and breed predispositions is available. Only a few case reports show breed and family relation in feline allergy, e.g., in the Abyssinian [21–23] and Devon Rex [24]. Further bias in feline studies is caused by the fact that the intact “barn cats” are rarely taken to a veterinarian, let alone to a specialist, to diagnose the disorder. A multicentric study, however, reported a predisposition for atopic dermatitis (non-flea/non-food hypersensitivity dermatitis [HD]) in young-adult, purebred and female cats. More precisely, 59% of 161 cats with atopic dermatitis were females [22]. Another Australian retrospective study of 45 atopic cats resulted in no gender predisposition when compared to the animal population at the university hospital [24]. The eosinophilic granuloma complex, which in felines is not identical with hypersensitive skin disease, was investigated in 17 Norwegian wildcats – 6 males (2 intact and 4 castrated) and 11 females (20 intact and 1 spayed) [25]. Atopic dermatitis and allergies were underlying the eosinophilic granuloma complex only in some of these cats, but gender effects were not reported. Collectively, to this day it has been impossible to conclude whether female, male or neutered cats are more frequently afflicted with allergies (especially atopic dermatitis) or with the eosinophilic granuloma complex. In a recent prospective clinical trial with 800 shelter kittens that focused on the investigation of the influence of early (at the age of 8–12 weeks) and late (at the age of 6–8 months) gonadectomy on different diseases (including dermatologic conditions), no statistical difference was observed as regards the development of these different diseases [26]. In this study, all kittens were adopted shortly after being neutered and then followed-up until 24 months of age. ^1^Speaking of feline asthma or feline bronchial disease, few studies with some contradictory results of sexual status on the disease are published. Female predispositions have previously been reported [27, 28], yet in a study by Fosters and others there was no sex predisposition noted [29].

## Gender differences related to allergies in horses: More research needed

Despite the clinical similarities of atopic dermatitis in horses and other pets, the equine AD remains poorly described. The main symptom seems to be itching and clinical symptoms appear seasonally or throughout the year.

Currently, only three skin diseases in horses are being differentiated in veterinary dermatology: atopic dermatitis (environmental allergy), food allergy and allergy to insect bites.

Recurrent airways obstruction, also known as heaves, is a chronic respiratory tract disease of horses characterized by coughing, mucopurulent airway exudate, increased respiratory efforts, and exercise intolerance. It is believed to be an allergy to inhaled molds and shows similarities to certain forms of asthma in humans.

Horses can be kept as a female horse (mare), male gelded horse (gelding) and male intact horse (stallion). As equine atopic dermatitis has rarely been described in clinical studies, only little is known about the gender influence. In one retrospective study on atopic dermatitis in horses with 54 animals, male horses (predominantly geldings) were over-represented in contrast to their female counterparts (35:19). However, this is statistically insignificant compared to gender distribution in the clinical study population [30]. Currently, therefore, there are no reliable data that could provide information about gender predisposition in atopic horses.

In a recently published study on insect bite hypersensitivity (IBH) in the Old Grey Kladruber horse, data on 1209 studbook horses were recorded and analyzed over a period of 13 years (1996–2009). There was a smaller risk of affliction in the stallions than in the mares and this difference was statistically significant [31]. In an epidemiological study on summer eczema (SE) in 490 Icelandic horses from 24 stud farms, mares (33.1%) and geldings (29.1%) were more often affected by SE than stallions 15.5% [32]. Another retrospective case-control study investigated 1.444 horses with recurrent airway obstruction (RAO) and 1444 control horses (examined for other reasons). The risk of RAO in females was 1.4 times higher than in sexually intact males (*P* = 0.004), and females were more likely to be examined for RAO than sexually intact males after adjusting for breed and age [33]. On the contrary, previous studies did not identify sex differences as regards susceptibility to RAO. The biological explanation for this difference is unclear, but it is possible that some of the genetic traits predisposing horses to RAO are sex-linked, or that environmental exposures of females (e.g., broodmares) are different from the environmental exposures of males.

## Discussion

In general, information about the influence of sex hormones in allergic disorder in companion animals is very limited. A recent study evaluating the neuter status and the sex of dogs with AD found a higher risk of disease development in neutered individuals (males and females) and in neutered female dogs when compared to neutered male dogs. The impact of sexual hormones on canine, feline or equine allergic diseases is an interesting research field and can help in finding comparative aspects in relation to human allergies, due to the neutering performed in companion animals. Therefore, more data are needed to investigate this field. The foundation of central databases, where research centers or universities would document and share their patients’ records would be very helpful. In North America, a central database collecting data of volunteering universities and centers of veterinary patients does exist (veterinary medical databases, VMDB), but it is not freely accessible.

Additionally, central state registers of all small animals (dogs and cats) and possibly horses, documenting species, date of birth, breed, gender, status/age of neutering, and diseases would be the next useful step in understanding hormonal influences. Data from commercial veterinary laboratories performing allergy testing may also – to a certain degree – be valuable. These institutions usually collect a complete signalment including the status of neutering and a rather detailed history.

In addition, longitudinal studies that would follow the relationship between the neutering of allergic dogs or cats and the amount of medication needed to control their AD might, to some degree, address our question.

## Conclusion

The main problem considering the correct evaluation of gender effects on atopic and/or allergic diseases in cats, dogs and horses is the lack of a central registry/database inhibiting the analysis of large data sets. The often practiced castration of domestic animals at different ages represents another complexity in approaching this question. Overall, more evidence is needed to document clinical importance of gender in allergies of domestic animals. Deeper knowledge could lead to practical recommendations for animal owners.
